# Correlation between neurofilament, HMGB1, MMP9, ds DNA blood levels and cognitive impairment in patients with neuropsychiatric systemic lupus erythematosus

**DOI:** 10.22088/cjim.15.1.6

**Published:** 2024

**Authors:** Arman Ahmadzade, Leila Simani, Mehrdad Roozbeh, Farane Farsad, Mehdi Sheibani, Omid Negaresh, Mohammad Mehdi Emam, Alireza Rajaei, Muhanna Kazempour, Mahtab Ramezani, Samad Nazarpoor

**Affiliations:** 1 Department of Rheumatology, Loghman Hakim Hospital, Shahid Beheshti University of Medical Sciences, Tehran, Iran; 2 Department of Pharmaceutical Sciences, College of Pharmacy, Kentucky University, Lexington, KY, USA; 3 Department of Neurology, Loghman Hakim Hospital, Shahid Beheshti University of Medical Sciences, Tehran, Iran; 4 Cardiovascular Research Center, Shahid Beheshti University of Medical Sciences, Tehran; 5 School of Medicine, Shahid Beheshti University of Medical Sciences, Tehran, Iran

**Keywords:** Systemic lupus erythematosus, Cognitive disorders, Neuropsychiatric systemic lupus erythematosus, Autoantibodies, Neuroinflammation.

## Abstract

**Background:**

Diagnosis of neuropsychiatric systemic lupus erythematosus (NPSLE) is challenging due to nonspecific biomarkers. High serum levels of neurofilament protein light subunit (NFL), high mobility group box 1 (HMGB1), Matrix metalloproteinase-9 (MMP-9) and have been reported in several autoimmune diseases. The aim of this study was to examine whether their plasma levels could serve as a diagnostic or prognostic biomarker for NPSLE.

**Methods:**

There were 90 SLE patients enrolled in this cross-sectional study (87.8% women and 12.2% men with a mean age of 41.67±11.05 years). We assessed the mental status of patients, also we measured the Systemic Lupus Erythematosus Disease Activity Index 2000 (SLEDAI-2K) and Systemic Lupus International Collaborating Clinics/ACR (SLICC/ ACR) Damage Index or SDI scores. Serum levels of NFL, HMGB1, MMP9, and ds-DNA were investigated to find a role in the pathophysiology of NPSLE.

**Results:**

Among the 90 patients with SLE, 63 (70%) met the criteria of NPSLE syndrome. Our results have shown a notable difference concerning SEDIAC-2k score, SDI score, PANS, MoCA, and Beck anxiety depression, between the two groups (p < 0.05). Although serum level of all measured serum biomarkers (NFL, MMP-9, HMGB1, dsDNA) were higher in patients with NPSLE, the difference was not statistically significant. Interestingly, our results showed that the serum level of NFL was correlated with the serum level of HMGB-1 and MMP-9. (r: 0.411, P=0.003).

**Conclusion:**

Serum level of NFL, HMGB-1 and MMP-9 may be used to detect abnormal mental status in patients with SLE.

Systemic lupus erythematosus (SLE) disease, as part of its autoimmune nature, can also affect the nervous system, referred to as neuropsychiatric systemic lupus erythematosus (NPSLE) (1). According to an ACR subcommittee, there are 19 types of NPSLE, including 12 CNS and seven PNS syndromes (1), with a prevalence ranging from 6% to 91% (2). The absence of specific and sensitive laboratory tests to confirm BBB dysfunction makes it challenging for rheumatologists to diagnose NPSLE (3). Both neuroinflammatory and ischemic mechanisms are implicated in the pathogenesis of NPSLE. Neuropsychiatric SLE can be challenging to diagnose; neither imaging nor laboratory studies are sensitive or specific enough to confirm the diagnosis (3). Neuropsychiatric events have negative impact on the quality of life in these patients (3). 

Among SLE neuropsychiatric manifestations, cognitive impairment (CI) is occasionally problematic to diagnose (4), with a prevalence of 14-88 percent, depending on the disease spectrum, neurocognitive tests used, and impairment definition (5) . More than half of SLE patients experiencing overt NP problems such as strokes or seizures suffer from CI (5, 6). SLE patients with isolated cognitive deficits approximately 30% of the population (5). 

Cognitive and emotional disturbances in SLE may be due to cross-reaction of antibodies with the N-methyl-D-aspartate subunits GluN2A/GluN2B, leading to disruption of the BBB, and resulting in apoptotic, non-inflammatory cell death in the CNS (7-10). It is problematic to interpretation of whether the CI is due to an incurable, chronic disease or a direct physiopathological mechanism associated with SLE, in clinical practice, particularly with milder manifestations of these conditions (11).To make a better diagnosis and treatment of NPSLE, it is necessary to identify more specific biomarkers.

As a structural component of neurons, neurofilament triplet protein light subunit (NFL) serves as a biochemical marker, especially in regard to axonal damage (12-14). Prior studies have revealed that a number of neurologic and even psychiatric diseases show increased level of serum NLF (15), additionally a high level of NFL in the CSF is associated with the presence of CNS inflammation in primary Sjogren's syndrome, SLE, and multiple sclerosis (14, 16). Moreover, in SLE patients, serum NLF (sNfL) has been reported to be a biomarker of neuropsychiatric involvement (17-19). According to previous data, patients with SLE have a high level of serum high mobility group box 1 (HMGB1), suggesting this protein is a marker for active disease (20, 21). In addition, Matrix metalloproteinase-9 (MMP-9) levels have elevated in CSF and serum of NPSLE patients with CI in particular(22, 23). Increased CSF levels of MMP-9 have also been correlated with neuronal and glial degradation markers in SLE patients, suggesting a link between MMP-9 production and CNS damage (23).

As biomarkers in peripheral blood can be identified and validated for NPSLE, the main objective of our study is to examine the relationship between serum levels of NFL, HMGB1, MMP9, and anti-dsDNA as a specific antibody and available test for SLE follow-up, focusing on patients with neuropsychiatric involvement (anxiety, mood disorders, psychosis, and cognitive impairment). Moreover, we aimed to analyze the mentioned serum biomarker levels in patients with and without neuropsychiatric manifestations. To our knowledge, few studies have been conducted on this subject.

## Methods

**Research design and ethics statements:** The Ethics Committee of Shahid Beheshti University of Medical Sciences, Tehran, Iran, has approved this study. (IR.SBMU.RETECH.REC.1399.654). 

**Participants and clinical examination**: In this cross-sectional study, conducted from November 2020- October 2021, we prospectively enrolled 90 SLE patients who were previously diagnosed with SLE at the rheumatology clinic of Loghman Hakim Hospital, Tehran, Iran. Inclusion criteria include being over 16 years old and meeting four of the American College of Rheumatology's (ACR) revised classification criteria (24). Hospitalized patients and patients with acute confusional states or infections were excluded. Demographic data such as age, gender, marital status, education level, the first presentation of SLE, disease duration, and a detailed history of medication including all currently and recently prescribed drugs have been assessed. Sapporo Criteria were used to diagnose antiphospholipid syndrome (APS) (25). 

A rheumatologist and a neurologist interviewed all patients to confirm the diagnosis and assessed the Systemic Lupus Erythematosus Disease Activity Index 2000 (SLEDAI-2K) (Range 0–105) and Systemic Lupus International Collaborating Clinics/ACR (SLICC/ ACR) Damage Index or SDI scores (Range 0–46)(26, 27). 

**Cognitive assessments: **All patients completed a self-administered questionnaire, which addressed neuropsychiatric manifestations including headache, seizure, cerebrovascular disease, mood disorder, cognitive dysfunction, anxiety, psychosis, movement disorder, acute confusional, aseptic meningitis, myelopathy, and multiple sclerosis-like syndromes. Neuropsychological assessment was performed by Montreal Cognitive Assessment (MoCA), Beck depression (BDI-II), Beck anxiety (BAI), and positive and negative syndrome scale (PANS).

**Montreal Cognitive Assessment (MoCA)**: In the MOCA, cognitive abilities are assessed, such as memory, visuospatial cognitive capacity, attention, concentration, executive functions, language, abstraction, orientation and calculation. It has a 30- point score (28). The score less than the standard cut-off of 26 points gives meaning to the presence of MCI (29, 30).

**Beck Depression Inventory- II (BDI-II)**: is self-report inventory measuring depression severity by using 21 items. (Range 0-63). (31, 32).

**Beck Anxiety Inventory (BAI): **A self-administered anxiety scale measuring 21 common somatic and cognitive symptoms (Range 0-63) (33, 34).

**The Positive and negative syndrome scale (PANSS)**: questionnaire is a medical scale for the evaluation of antipsychotic treatment, which consists of 30 questions (Range 30–150) (35, 36).

Based on NPSLE2 definition (2) (SLE and at least one altered cognitive and/or neuropsychiatric status manifestation or sensorimotor neuropathy) all participants were classified into two groups: NPSLE (N=63) and Non-NPSLE (N=27) patients. 

**Laboratory measurement:** Peripheral venous blood was sent for routine laboratory tests (including complete blood counts, CRP, ESR, C3, C4, renal function test, TSH). In addition, five ml of venous blood were collected at the time of the examination and properly stored. Blood was kept at room temperature for 20 minutes to allow coagulation to complete. After centrifugation at 1,000G for 10 minutes, serum was separated. We investigated serum levels of NFL, HMGB1, MMP9 and dsDNA by ELISA (enzyme-linked immunosorbent assay) levels by using SUNRISE, a new microplate reader from TECAN.

**Statistical analysis:** The analysis was performed using a statistical package for the social sciences (SPSS, Version 18). The Kolmogorov-Smirnov test was used to assess variable normal distribution. The numerical and categorical variables were expressed as mean ± standard deviation frequency and percentage, respectively. The independent sample t-test, chi-square, and Mann-Whitney test were applied to analyze differences between variables in quantitative and categorical data. In addition, Spearmen’s correlation was performed to explore the association between biomarkers level with SLE characteristics (i.e., anxiety, depression, psychosis, CNS complication, medication, etc.). A p-value below 0.05 was considered statistically significant.

## Results

In this cross-sectional study, 90 patients with SLE diagnosis were enrolled (87.8% females and 12.2% males with a mean age of 41.67±11.05 years). The mean disease duration was 10.28±8.26 years. Regarding major risk factors of comorbidity, 19 (21%) patients had a history of hypothyroidism and 7 (7.8%) patients had diabetes mellitus and cardiovascular disease, respectively.

Among the 90 patients with SLE, 63 (70%) met the criteria of NPSLE syndrome. Neither group showed significant demographic differences (i.e., sex or age). (p > 0.05), (table 1).

 However, our study showed a notable difference concerning SEDIAC-2k score, SDI score, PANS, MoCA, and Beck anxiety depression), between the two groups (P < 0.05). In addition, in terms of CNS manifestation self-questionnaire, 52 patients (82.5%) in NPSLE group suffered from cognitive dysfunction, 37(58.7%) mood disorder, 14 (22.2%) seizure, 9(14.3%) cerebrovascular disease, and 10(15.9%) psychosis. Patients with NPSLE had significantly more CNS manifestation than non-NPSLE (p<0.05), (table 1).

**Table 1:** Characteristics and Clinical features of study groups

**Variables**	**Total** **(N=90)**	**NPSLE** **(N=63)**	**Non-NPSLE (N=27)**	**P-value**
Sex Male/Female	11(12.2)/79(87.8)	8(12.7)/ 55(87.3)	3(11.1)/24(88.9)	0.833
Age (years)	41.67±11.05	42.20±11.25	40.44±10.69	0.492
Disease duration	10.28±8.26	10.17±8.31	10.51±8.28	0.859
SLEDAK-2K scores	11.47±8.9	12.66±9.36	8.70±7.14	0.032
SDI scores	2.05±2.02	2.44±2.13	1.14±1.4	0.001
**Comorbidity** Hypothyroidism HTN DM	19(21) 7(7.8) 7(7.8)	15(23.8) 7(11.1) 6(9.5)	4(14.8) 0 1(3.7)	0.338 0.071 0.345
APS syndrome	17(18.9)	12(19)	5(18.5)	0.953
MoCA	21.16±5.51	19.15±5.34	25.85±1.87	0.001
Beck anxiety	20.18±13.62	22.61±13.67	14.51±11.9	0.009
Beck depression	19.23±12.78	21.34±12.87	14.29±11.33	0.016
PANS	59.95±19.7	62.82±19.73	53.25±18.26	0.034
**History of CNS Manifestation** Headache CVD Seizure Mood disorder Cognitive dysfunction Anxiety psychosis Movement disorder Acute confusion Aseptic meningitis Myelopathy MS like syndrome	37(41.1) 9(10) 15(15.6) 43(47.8) 62(68.9) 50(55.6) 10(11.1) 2(2.2) 2(2.2) 1(1.1) 3(3.3) 4(4.4)	25(37.9) 9(14.3) 14(22.2) 37(58.7) 52(82.5) 37(58.7) 10(15.9) 2(3.2) 2(3.2) 0 3(4.8) 3(4.8)	12(44.4) 0 0 6(22.2) 10(37) 13(48.1) 0 0 0 1(3.7) 0 1(3.7)	0.674 0.038 0.008 0.001 0.001 0.355 0.028 0.349 0.349 0.125 0.249 0.823
**Medication** Prednisolone Hydroxychloroquine Mycophenolate Azathioprine Methotrexate Tacrolimus Cyclosporine Rituximab Cyclophosphamide ASA. Clopidogrel Warfarin Rivaroxaban/Apixaban	71(78.9) 70(77.8) 20(22.2) 29(32.2) 7(7.8) 15(16.7) 1(1.1) 6(6.7) 2(2.2) 30(33.3) 7(7.8) 5(5.6)	51(81) 49(77.8) 19(30.2) 16(25.8) 6(9.5) 14(22.2) 1(1.6) 6(9.5) 2(3.2) 21(33.3) 6(9.5) 4(6.3)	20(74.1) 21(77.8) 1(3.7) 13(48.1) 1(3.7) 1(3.7) 0 0 0 9(33.3) 1(3.7) 1(3.7)	0.464 1.00 0.006 0.039 0.345 0.035 0.25 0.097 0.349 1.00 0.345 1.00
Anti-depressant Anti-convulsant Anti-psychotic	16(17.8) 9(10) 3(3.3)	10(15.9) 9(14.3) 3(4.8)	6(22.2) 0 0	0.470 0.09 0.249
^* ^N (%);^ ^ ^Mean±SD; P<0.05				

NPSLE: Neuropsychiatric systemic lupus erythematosus, SLEDAK-2K: Systemic Lupus Erythematosus Disease Activity Index 2000, SDI: Systemic Lupus International Collaborating Clinics/American College of Rheumatology (SLICC/ACR) Damage Index, HTN: Hypertension, DM: Diabetes mellitus, APS: Antiphospholipid syndrome, MoCA:  Montreal Cognitive Assessment, PANS: Positive and Negative Syndrome Scale, CNS: Central nervous system, CVD: Cardiovascular diseases, ASA: acetylsalicylic acid

Although serum level of all measured serum biomarkers was higher in patients with NPSLE, following the t-test, no significant differences were observed in levels of NFL, MMP-9, HMGB1, dsDNA, CRP, and ESR between the two groups (p > 0.05) (table 2). Moreover, regarding the association of CNS manifestation, SEDIAC-2k scores, and SDI with sNfL level, the variables were not statistically correlated. Interestingly, we found a negative correlation between SDI scores and serum levels of HMGB-1 (r: -0.39, P =0.044) in patients with non-NPSLE. Additionally, there was a reverse relation between anxiety and PANS and the level of MMP-9 (r: -0.344, P=0.02; r: -0.309, P= 0.026, respectively) in patients with NPSLE. A positive correlation was detected between SEDIAC-2k scores and dsDNA in patients with non-NPSLE (r: 0.622, P=0.001). According to our data, serum biomarkers are not statistically significantly affected by different kinds of medications.

The serum level of NfL was correlated with the serum level of HMGB-1 and MMP-9 (r: 0.411, P = 0.003). Moreover, a positive relation was detected between HMGB-1 and MMP-9 in two groups. (r: 0.589, P = 0.001 for positive; r: 0.402, P = 0.038 for negative).

**Table 2:** Mean ±SD level of biomarkers

**Variables**	**Total** **(N=90)**	**NPSLE** **(N=63)**	**Non-NPSLE (N=27)**	**P-value**
**NFL**	7.75±14.65	7.96±15.97	7.26±11.32	0.294
**HMGB-1**	27.89±4.54	27.98±3.51	27.72±6.14	0.236
**MMP-9**	1649.34±744.46	1729.06±817.54	1495.80±560.75	0.996
**dsDNA**	139.76±163.20	141.93±169.11	134.71±151.46	0.676
**ESR**	19.25±15.08	20.11±16.15	17.26±12.32	0.425
**CRP**	2.04±1.44	2.1±1.73	1.92±0.27	0.607

NFL: Neurofilament, HMGB-1: High mobility group box 1, MMP-9: Matrix metalloproteinase-9, dsDNA: double stranded DNA, ESR: Erythrocyte sedimentation rate, CRP: C-reactive protein

## Discussion

Diagnosis of NPSLE and confirmation of the CI in patients with SLE can be challenging and more complicated, especially in individuals with mild manifestations. Our results revealed that SLE patients who were considered NPSLE have shown a lower MoCA score as well as higher anxiety, depression, and positive and negative symptoms of schizophrenia in comparison with the non-NPSLE group. Prior studies have demonstrated that NPSLE patients consistently showed lower cognitive performance specifically in attention, memory, executive function, and visual coordination (37). Considering clinical factors associated with CI in SLE, the study was conducted by Gao, Y. et al. revealed that there was a higher risk of cognitive dysfunction among patients who had a longer duration of SLE, pre-existing cerebral involvement, and multiple medical complications (38). However, we found no significant differences regarding these factors in our patients. Moreover, although the vascular risk factor and disease activity are the two accused of CI in NPSLE, other neuropathology involving the immune cells, cytokines, chemokines, and autoantibodies (Abs), have been suggested (39).

On the other hand, based on previous data, depression and anxiety are two main cofounders that affect cognitive performance in SLE patients (40, 41). Ho. et al. in their study, found that participants with high levels of anxiety/depression significantly slowed processing speeds compared to patients with low levels of anxiety/depression (42). Because of a genetic vulnerability attributed to the level of serotonin and inflammation in depression and CI, these two entities may share the same underlying pathophysiological mechanisms (43, 44). In addition, cognitive dysfunction and depression are associated with high levels of IL-6 as a pro-inflammatory cytokine (45). Therefore, a significantly lower score on the MOCA test in our NPSLE patients may be due to a higher level of anxiety or depression. 

Although our results showed no notable differences regarding the levels of NFL, MMP-9, HMGB1, and dsDNA, between the two groups, a positive correlation was detected between HMGB-1 and MMP-9, and NFL in both groups. In NPSLE, HMGB1 can be secreted by stressed or activated cells following disruption of the blood-brain barrier (46). HMGB1, as a nuclear protein has been identified to mediate inflammation when released from necrotic cells, which can affect both innate and adaptive immune systems, leading to SLE disease progression (47). Moreover, Aragón, CC. et al. suggested that HMGB1 proteins may serve as biomarkers for disease progression (48).

MMP-9 is a family of zinc-dependent enzymes involved in many inflammatory and autoimmune processes (49). There are discrepancies in the published data regarding the role of MMP-9 in SLE. Some evidence has found that MMP-9 serum levels are higher in SLE patients than in healthy controls, while other authors fail to detect such a significant difference (50-52).

Serum NfL (sNfL), as a marker of neuronal cytoskeleton, has shown to increase in the cerebrospinal fluid (CSF) of several neurodegenerative disorders as well as autoimmune inflammatory diseases, including multiple sclerosis, SLE, and primary Sjøgren’s syndrome (17, 53). A recent study has identified some abnormal cognitive, neurological and neuroimaging findings associated with elevated sNfL levels in NPSLE individuals; however, the authors concluded that NfL in CSF appears to be a better marker of neuronal damage in SLE patients (18). 

The elevated levels of mentioned serum markers in our study are in line with previous research, although we found no differences between the two groups. That is because we have evaluated a small number of SLE patients without any major or active NP manifestation; additionally, the CSF level of NfL may have a better yield in comparison to the two groups of SLE patients. Engel, S. et al. in their study in 2021, also suggested that sNfL concentration only increased in NPSLE with focal cerebral lesion compared to the non-NPSLE group (19).

Our study has several limitations. None of our patients have major NP manifestations; additionally, we did not evaluate the patient's specific aspects of cognition at the survey time. Therefore, a further study with a larger sample size, including clinically diverse groups, along with an evaluation of the CSF level of markers should be accomplished. Whereas serum level of NFL correlated with the serum level of HMGB-1 and MMP-9, serum level of these biomarkers may be used to detect abnormal mental status in patients with SLE.
